# Diagnostic Reference Levels for nuclear medicine imaging in Austria: A nationwide survey of used dose levels for adult patients

**DOI:** 10.1016/j.zemedi.2021.11.007

**Published:** 2022-01-20

**Authors:** David Wachabauer, Thomas Beyer, Manfred Ditto, Hans-Jürgen Gallowitsch, Michael Hinterreiter, Bettina Ibi, Phillipp Malle, Siroos Mirzaei, Florian Smetana, Anton Staudenherz, Boris Warwitz, Georg Zettinig, Ivo Rausch

**Affiliations:** a Austrian National Public Health Institute (Gesundheit Österreich GmbH (GÖG)), Vienna, Austria; b QIMP Team, Center for Medical Physics and Biomedical Engineering, Medical University Vienna, Austria; c Austrian Society of Nuclear Medicine and Molecular Biology (OGNMB), Vienna, Austria; d Federal Ministry of Social Affairs, Health, Care and Consumer Protection, Vienna, Austria; e Department of Nuclear Medicine and Endocrinology, Klinikum Klagenfurt am Wörthersee, Klagenfurt, Austria; f rtaustria - Austrian Society of Radiological Technologists, Wiener Neustadt, Austria; g Clinic Hietzing, Institute for Hospital Physics, Vienna, Austria; h Austrian Society for Medical Physics (ÖGMP), Vienna, Austria; i Institute of Diagnostic and Interventional Radiology, Klinikum Klagenfurt am Wörthersee, Klagenfurt, Austria; j Austrian Roentgen Society, Vienna, Austria; k Karl Landsteiner University of Health Sciences; Department of Nuclear Medicine, University Hospital St. Poelten, Austria; l Austrian Society for Radiation Protection in Medicine (VMSÖ), Vienna, Austria; m Department of Nuclear Medicine, Medical University Innsbruck, Innsbruck, Austria; n Vienna Thyroid Center Schilddruesenpraxis Josefstadt, Vienna, Austria; o Federal Working Group Nuclear Medicine of the Austrian Medical Association, Austria

**Keywords:** BGO, Bismuth Germanate, CT, computed tomography, CZT, cadmium-zinc-telluride, DRL, diagnostic reference levels, EU, European Union, FOV, field of view, HTML, hypertext markup language, ICRP, International Commission on Radiological Protection, IR, iterative reconstruction, LSO, Oxyorthosilicate, NaI, Natriumiodide, NDRL, national diagnostic reference levels, NUC, nuclear medicine imaging, PET, positron emission tomography, SD, standard deviation, SPECT, single photon emission computed tomography, TOF, time-of-flight, Radiation Protection, Nuclear medicine, Diagnostic reference levels

## Abstract

**Purpose:**

To assess dose levels in routine nuclear medicine (NUC) procedures in Austria as a prior to a legislative update of the National Diagnostic Reference Levels (NDRL).

**Method:**

As part of a nationwide survey of common NUC-examinations between June 2019 and November 2019, data sets were collected from 33 Austrian hospitals with NUC equipment. All hospitals were asked to report the NUC imaging devices in use (model, type, year of manufacture, detector material, collimators), the standard protocol parameters for selected examinations (standard activity, collimator, average acquisition time, reconstruction type, use of time-of-flight) and to report data from 10 representative examinations (e.g. injected activity, weight), incl. the most common NUC-examinations for planar imaging/SPECT and PET. Median/mean values for injected activity were calculated and compared to current Austrian and international NDRL. A Pearson correlation coefficient was computed comparing different variables.

**Results:**

In total, all 33 hospitals (100% response rate) reported data for this study for 60 SPECT devices, 21 PET/CT devices and 23 scintigraphy devices. Fixed activity values for scintigraphy/SPECT and PET were employed by about 90% and 56% of the hospitals, respectively. The most widely performed examinations for scintigraphy/SPECT are bone imaging, thyroid imaging, renal imaging (with MAG3/EC) and lung perfusion imaging (in 88% of the hospitals) and F-18 FDG-PET studies for oncology indications (in 100% of the hospitals). Significant correlations were found for patient weight and injected activity (scintigraphy/SPECT), use of iterative reconstruction and injected activity (PET) as well as size of field-of-view and injected activity (PET).

**Conclusions:**

The reported injected activity levels were comparable to those in other countries. However, for procedures for which NDRL exist, deviations in injected activities of >20% compared to the NDRL were found. These deviations are assumed to result mainly from advances in technology but also from deviations between NDRL and prescribed activities as given in the information leaflets of the radiopharmaceuticals.

## Introduction

Over the past century, medical imaging has evolved to an indispensable part of diagnostic procedures. However, some of the most common imaging methods, such as X-ray, computed tomography (CT) as well as nuclear medicine imaging (NUC) procedures, such as scintigraphy, single photon emission computed tomography (SPECT) and positron emission tomography (PET) make use of ionizing radiation, and, thus, inherently imply radiation exposure to patients [1]. Even though the exposure from diagnostic procedures is small (in the range of 0.01 mSv to 20 mSv effective dose) and eventual negative effects of such small exposures are controversially discussed [2], [3], [4], in sum they substantially contribute to the general radiation exposure of the population [1], [5], [6].

To limit the general radiation exposure of the population and potential adverse effects to individual patients, diagnostic procedures including ionizing radiation need to follow the ALARA (As Low As Reasonable Achievable) principle. Thus, exposure from medical imaging should be limited to the lowest level necessary to reliably answer the respective diagnostic question. To achieve this, diagnostic procedures should be continuously optimized, among others considering technical advances in imaging technologies and changes in diagnostic practice.

To monitor and facilitate the dose optimization process, the International Commission for Radiologic Protection (ICRP) introduced the concept of diagnostic reference levels (DRL) [7], [8], [9]. DRL are standard levels of easily measurable quantities, such as the dose length product in CT or injected activity in NUC, for common procedures. DRL set a general guidance for the dose in clinical operation and help to identify if routine doses are unusually high or low. They do not apply directly to individual examinations and patients [7].

According to the recommendations of the ICRP [7] and European legislation [10], DRL are set and updated on national level based on the assessment of observed dose distributions. Therefore, national radiological and NUC procedures need to be regularly surveyed to account for changes in diagnostic practice and technological advances.

In the standard literature, two main approaches for setting national diagnostic reference levels (NDRL) in NUC are discussed: first, as outlined in Radiation Protection Radiation Protection N° 109 [11] of the European Commission, NDRL in NUC are administered doses necessary for a good image during a standard procedure, which follow the concept of “optimal doses”. The DRL should be approached as closely as possible and a substantial deviation of these optimal doses is only permitted for special occasions, such as for obese patients. Consequently, following this concept, NDRL for NUC are not set at the third quartile but rather at the median or mean of each examination type and these recommendations should be developed in close cooperation with relevant societies and experts. This concept is implemented in Austria [12] or Switzerland [13] and was recommended by the European Commission in 2014 [14]. Another approach would be to use the same definition and system for DRL as it is valid for CT or radiography. Consequently, NDRL are determined based on the third quartile of a dose distribution. If these NDRL are on average constantly exceeded, justification is necessary. The ICRP 103 [7] recommends using this approach and several publications were recently published [15], [16], [17]. Also a combination of these two outlined approaches is possible and was described by A Shahzad, S Bashir and A Anwar [18].

In Austria, NDRL are regulated in the Austrian Medical Radiation Protection Ordinance [12], [19]. For NUC, this ordinance states “Diagnostic reference levels for nuclear medicine examinations are defined as activities to be administered to standard-sized adult persons.” [12] and a deviation from the NDRL by more than 20% must be justified. Consequently, in the course of this study, NDRL were developed based on the median (and mean) values in close cooperation with NUC experts and under involvement of all relevant societies. Additionally, third quartiles of the dose distribution are provided for comparison with other NDRL derived based on the third quartile.

The last evaluation of diagnostic procedures and respective doses of NUC examinations in Austria was performed in 2008 [20], [21]; the NDRL were updated in 2010 accordingly [19]. Since then, significant technological advances in imaging technologies have been made and new radiopharmaceuticals have come to use. Technical advances include the introduction of direct photon detection with cadmium-zinc-telluride (CZT) based detectors for scintigraphy and SPECT imaging [22]. For specific organ examinations, such as myocardial perfusion imaging, dedicated SPECT devices and optimized collimators are more commonly available now [23]. In PET imaging, a general trend to extended axial field-of-views (FOV), and, thus, improved sensitivity can be seen [24], [25]. Furthermore, the implementation of semiconductor-based PET detectors and improved detection electronics led to significant advances in time-of-flight (TOF) resolution, and, in turn, improvements in signal-to-noise ratios [26]. Finally, progress in image reconstruction has been made within the last decade for SPECT as well as PET imaging [27], [28].

In parallel to the technical development, new diagnostic agents and respective procedures were established. For example, in the last decade Tc-99m- and Ga-68 labelled PSMA was introduced for the assessment of prostate cancer [29]. F-18 labelled amyloid tracers for dementia imaging are now commonly used [30]. F-18 labelled choline has been established for parathyroid imaging [31] and cardiac SPECT examinations shifted from mainly based on thallium-chloride to the use of technetium labelled perfusion tracers [32].

The Federal Ministry of Health and the Austrian National Public Health Institute engage in updating the Austrian NDRL for medical imaging procedures [33], [34]. To assess these above-mentioned changes in technology and clinical practice and to update the enforced NDRL for NUC, a survey of NUC procedures was performed across Austria. This included the assessment of the used technology, examination protocols and administered activities. Here, we report on the methodology and the outcome of this survey which was the basis of the update of the NDRL for NUC in Austria.

## Material and methods

The study design of this nationwide survey was conducted with reference to similar international studies and based on the recommendations by the ICRP 135 [7].

### Examination types

To define relevant examination types for the survey, Austrian NDRL for NUC (which were enforced at that time) were used [19]. In addition, the last study on Austrian NDRL for NUC by A Stemberger, T Leitha and A Staudenherz [21], a report by the European Commission [14] on European NDRL, NDRL from Switzerland [13] and Germany [35] were included and discussed with a multidisciplinary expert group consisting of radiologists, medical physicists and radiographers as well as representatives from relevant societies. Frequencies of relevant NUC-examinations were taken from the Austrian-wide routine documentation data held and maintained by the Austrian Federal Ministry of Health [6], [36]. Table 1 and Table 2 depict the examination types and indications included into the survey besides obligatory and optional parameters, respectively.

**Table 1:** Types of planar scintigraphy imaging/SPECT examinations as well as mandatory and optional survey parameters.

Procedure	Radionuclide	Imaging agent	survey parameters	
			Mandatory	optional
Brain imaging	I-123	I-123 benzamide, beta-CIT	a) Per device: • Manufacturer • Model • Year of Manufacture • Type [scintigraphy device, SPECT, SPECT/CT] • Detector material [NaI, CZT]	
Salivary gland scan	Tc-99m	Tc-99m pertechnetate		
Thyroid imaging	Tc-99m	Tc-99m pertechnetate		
Thyroid whole body imaging	I-131	I-131 sodium iodide		
Parathyroid imaging	Tc-99m	Tc-99m isonitrile		
Myocardial perfusion imaging (rest and stress)	Tc-99m	Tc-99m isonitrile; 1-day and 2-day protocol		
Myocardial perfusion/vitality imaging	Tl-201	Tl-201 chloride		
Lung perfusion imaging	Tc-99m	Tc-99m macroaggregates	b) Per examination protocol: • Activity [MBq], fixed or per kg • Collimator [LEHR, LEGP, MEGP, HEGP, other]	b) Per examination protocol: • Acquisition time per patient [min]
Gastric imaging	Tc-99m marked chyme	Tc-99m marked chyme colloid		
Renal imaging	Tc-99m	Tc-99m MAG3/EC		
Renal imaging	Tc-99m	Tc-99m DMSA		
Renal imaging	Tc-99m	Tc-99m DTPA		
Adrenal imaging	I-123	I-123 MIBG		
Bleeding	Tc-99m	Tc-99m pertechnetate, erythrocytes		
Inflammation imaging	Tc-99m	Tc-99m anti-granulocytes antibodies		
SNL lymphoscintigraphy	Tc-99m	Tc-99m colloid – 1-day and 2-day protocol		
Bone imaging	Tc-99m	Tc-99m bisphosphonate		
Tumour/inflammation/receptor imaging	In-111	In-111 somatostatin receptor antagonist	c) Per patient: • injected activity [MBq] • Weight [kg]	c) Per patient: • Height • Sex • Year of birth
Tumour/inflammation/receptor imaging	Tc-99m	Tc-99m PSMA		
Tumour/inflammation/receptor imaging	Tc-99m	Tc-99m somatostatin receptor antagonist

**Table 2:** Types of PET examinations as well as mandatory and optional survey parameters.

Procedure	Radionuclide	Imaging agent	survey parameters	
			Mandatory	optional
Brain imaging	F-18	F-18 FDG	a) Per device: • Manufacturer • Model • Year of Manufacture axial FOV in cm (in Z-direction) • Detector material [LSO, BGO]	
Brain imaging	F-18	F-18 tyrosine		
Myocardial imaging	F-18	F-18 FDG		
Tumour imaging	Cu-64	Cu-64 PSMA	b) Per examination: • Activity [MBq], fixed or per kg • average acquisition time per bed position [min] • iterative reconstruction [yes, no] • Time of Flight [yes, no]	
Tumour imaging	F-18	F-18 choline		
Tumour imaging	F-18	F-18 FDG		
Tumour imaging	F-18	F-18 fluoride		
Tumour imaging	Ga-68	Ga-68 PSMA	c) Per patient: • injected activity [MBq] • weight [kg]	c) Per patient: • Height • Sex • Year of birth
Tumour imaging	Ga-68	Ga-68 somatostatin receptor antagonist		

### Survey design and data entry form

In Austria, approximately 39% of the NUC examinations are performed in private imaging centres (= ambulatory care sector) and 61% in hospitals (only including examinations which are fully covered by the social insurance system) [30]. The predominant examination in the ambulatory care sector, approx. 83%, are thyroid imaging examinations. The inclusion of private imaging centres would have caused practical issues by means of availability of contact data, thus, the decision was made that this survey will only include hospitals. We expect that the data collected is representative also for private imaging centres as the majority of examinations are performed in hospitals and thyroid imaging is a well standardised examination.

All 33 hospitals with NUC equipment were invited via e-mail, followed up by e-mail reminders and, if necessary, telephone calls to non-responding hospitals to increase response rates. The data collection period lasted from June 2019 to October 2019. The survey was carried out using a HTML based online data entry form. Consequently, this offered the possibility to identify individual data entries, create standardized reports and retrospectively trace and analyse each data entry.

The survey consisted of three parts; (a) the assessment of the imaging devices used, (b) the definition the local standard protocols and (c) the collection of individual patient data.

(a) First, each hospital was asked to declare the NUC imaging devices in use. This included the manufacturer, model and year of manufacture of the NUC-imaging devices, detector material and the axial FOV (for PET) (Table 1 and Table 2).

(b) For each device, the hospitals were asked to enter the standard protocol parameters for the selected examinations. This included the standard activity (fixed or per kg), the collimator as well as (for PET) the average acquisition time per bed position, use of iterative reconstruction (IR) and TOF (Table 1 and Table 2).

(c) For each examination type, the hospitals were asked to report data from 10 representative examinations. The required information was the injected activity and the weight of the patient. On an optional basis, the form allowed to report the height, sex and year of birth of the patient.

### Data quality

Measures were taken to increase response rates and improve data quality. To incentivise study participation, analyses comparing the median activity values of an individual participating hospital to the new NDRL recommendations were advertised and made available to the participating hospitals. During the data collection phase, a questions and answers hotline was installed to allow participating hospitals to directly call and talk to experts in order to assist with and answer questions regarding data entry. Automated pop-up warning messages were integrated into the data entry form to directly inform the participants on entries with highly implausible values. In such cases the software would prompt the user to check the dose entered. To further improve data quality, data entries were centrally monitored during the data collection period and hospitals entering implausible dose values were contacted in short order to clarify eventual faulty entries. After the data entry period ended, data were again checked for plausibility.

After this plausibility check, data cleaning was performed especially for the Tc-99m DMSA examination type as several patient datasets were entered including children. Consequently all datasets with patients younger than 18 years were excluded (for planar/SPECT 123 datasets and for PET 0 datasets). For datasets without the year of birth provided, patient weight was used as a proxy and all datasets with patient weight equal or lower 35 kg were excluded (for planar/SPECT 21 datasets and for PET 0 datasets). The number of patient datasets with weight between 35 kg and 70 kg was 1,403 (40% of all datasets) for planar/SPECT and 353 (34% of all datasets) for PET. Furthermore, for myocardial imaging, values for stress and rest examinations had to be reported. If only stress examination were reported for myocardial imaging and no rest value was available, these datasets were excluded (in total 5 protocol datasets and 62 patient datasets). 13 patient datasets for the examination type thyroid whole body imaging were excluded as they were performed for therapy purpose.

### Statistical analysis

All analyses were conducted with the statistical software package R (Version 3.3.1; The R Foundation, Free Software Foundation) and Microsoft Excel (Office 365, Microsoft). Median, third quartiles, mean, standard deviation (SD), maximum and minimum were calculated for each examination type. Pearson's correlation coefficient was calculated to analyse the association between different variables such as injected activity and year of manufacture of the devices. Significance was set at a P value of <0.01.

## Results

In total, all 33 hospitals (100% response rate) reported data for this study. 64% (N = 21) of the hospitals have a dedicated scintigraphy device (a scintigraphy device without a SPECT option, e.g. dedicated thyroid cameras) in operation, 94% (N = 31) of the responding hospitals are equipped with a SPECT or SPECT/CT device, and 55% (N = 17) of those hospitals with a SPECT or SPECT/CT device additionally operate a PET/CT. 58% (N = 18) of the hospitals with SPECT or SPECT/CT have more than one SPECT or SPECT/CT device in operation, whereas only 24% (N = 4) of the hospitals operating a PET/CT have more than one PET/CT device. Table 3 and 4 summarizes the number of hospitals per examination type.

**Table 3:** Planar imaging/SPECT - Number (N) of hospitals/devices, data for examination protocols and collected patient data.

Examination type	N hospitals/devices	Prescribed activities in the examination protocols mean/median		Reported injected activities from the collected patient data					
		for protocols with a defined total activity [MBq]	for protocols with a weight based prescription [MBq/kg]	Number of reported datasets	Weight mean [kg]	Injected activity mean & SD [MBq]	Injected activity median [MBq]	Injected activity 3rd quartile [MBq]	Injected activity Min-max [MBq]
Brain imaging: I-123 benzamide beta-CIT	21/28	177/185	2.4/2.4	222	78	181 ± 19	185	187	129-230
Salivary gland scan: Tc-99m pertechnetate	14/20	137/111	2.6/2.6	135	71	162 ± 75	157	186	71-434
Thyroid imaging: Tc-99m pertechnetate	29/38	73/74	1.0/1.0	321	75	76 ± 16	76	85	37-122
Thyroid whole-body imaging: I-131 sodium iodide	15/19	194/185	-	132	76	178 ± 89	185	186	74-371
Parathyroid imaging: Tc-99m isonitrile	22/34	470/400	4.9/4.9	249	76	465 ± 135	400	578	302-768
Myocardial perfusion imaging: Tc-99m isonitrile 1-day protocol (rest and stress)	20/23	total: 1090/1050	total: 12.75/12.75	180	81	1108 ± 165	1084	1141	731-1884
Myocardial perfusion imaging: Tc-99m isonitrile 2-day protocol (rest and stress)	4/5	595/550 per day	4/4	30	80	total: 1085 ± 376	total: 1000	1558	509-1585
Myocardial perfusion/vitality imaging: TI-201 chloride	7/9	103/100	3.0/3.0	80	79	129 ± 88	100	111	64-376
Lung perfusion imaging: Tc-99m macroaggregates	29/42	129/125	1/1	345	79	128 ± 39	126	150	21-387
Gastric imaging: Tc-99m marked chyme	15/21	49/40	0.5/0.5	165	71	51 ± 29	41	55	10-165
Renal imaging: Tc-99m MAG3/EC	29/43	103/100	1.2/1	331	75	101 ± 30	98	110	57-205
Renal imaging: Tc-99m DMSA	17/23	110/100	1.4/1.4	110	73	109 ± 37	98	109	59-185
Renal imaging: Tc-99m DTPA	2/4	170/170	-	20	78	222 ± 15	223	232	176-247
Adrenal imaging: I-123 MIBG	7/9	302/300	-	42	81	288 ± 106	260	370	185-450
Bleeding: Tc-99m pertechnetate, erythrocytes	9/16	625/600	8.1/8.1	116	71	565 ± 73	556	597	400-808
Inflammation imaging: Tc-99m anti-granulocytes antibodies	13/15	661/700	-	104	81	688 ± 121	700	757	408-925
SNL lymphoscintigraphy: Tc-99m colloid – 1-day protocol	23/37	89/94	-	284	74	123 ± 138	98	138	21-781
SNL lymphoscintigraphy: Tc-99m colloid – 2-day protocol	5/8	94/110	-	62	74	119 ± 53	136	160	22-219
Bone imaging: Tc-99m bisphosphonate	29/50	627/650	8.9/9.1	424	77	652 ± 102	663	727	402-985
Tumour/inflammation/receptor imaging: In-111 somatostatin receptor antagonist	3/6	173/185	2.2/2.2	60	73	172 ± 32	160	199	118-257
Tumour/inflammation/receptor imaging: Tc-99m somatostatin receptor antagonist	8/10	731/740	-	79	77	751 ± 61	740	759	525-911
Tumour/inflammation/receptor imaging: Tc-99m PSMA	3/3	618/600	-	14	87	635 ± 57	607	661	587-786

**Table 4:** PET - Number (N) of hospitals/devices, data for the examination protocols and collected patient data.

Examination type	N hospitals/devices	Prescribed activities in the examination protocols mean/median		Reported injected activities from the collected patient data					
		for protocols with a defined total activity [MBq]	for protocols with a weight based prescription [MBq/kg]	Number of reported datasets	Weight mean [kg]	Injected activity mean & SD [MBq]	Injected activity median [MBq]	Injected activity 3rd quartile [MBq]	Injected activity Min-max [MBq]
Brain imaging: F-18 FDG	13/16	179/185	2.6/2.6	150	75	199 ± 65	197	230	70-397
Brain imaging: F-18 tyrosine	7/9	252/250	2.6/2.6	78	76	271 ± 51	260	295	172-393
Myocardial imaging: F-18 FDG	9/11	275/275	3.9/4.0	93	84	297 ± 61	300	348	154-452
Tumour imaging: F-18 FDG	17/21	271/260	3.8/3.6	207	76	279 ± 58	272	317	99-476
Tumour imaging: Ga-68 somatostatin receptor antagonist	12/15	150/150	2.2/2.0	146	78	159 ± 36	162	182	53-259
Tumour imaging: Cu-64 PSMA	3/3	250/250	4/4	30	79	288 ± 48	274	297	183-395
Tumour imaging: F-18 choline	11/14	250/250	3.7/3.5	123	78	278 ± 55	272	316	152-388
Tumour imaging: F-18 fluoride	4/6	217/200	-	50	78	246 ± 98	248	337	104-425
Tumour imaging: Ga-68 PSMA	13/17	156/150	2.1/2.0	168	83	175 ± 37	173	189	111-330

**a) Devices:** In total, data was reported for 60 SPECT or SPECT/CT devices and for 21 PET/CT devices in operation. In addition, data were submitted for 23 scintigraphy devices. On average, scintigraphy devices were installed in 2009 (min: 1999; max: 2017), for SPECT or SPECT/CT devices 2010 (min: 2000; max: 2019), and for PET/CT devices 2013 (min: 2008; max: 2018). 100% (N = 23) of the scintigraphy devices as well as 97% (N = 58) of the SPECT or SPECT/CT devices use Natriumiodide (NaI) as a scintillator material whereas only 3% (N = 2) of the SPECT or SPECT/CT devices use CZT. 95% (N = 20) of the PET/CT devices use Lutetium Oxyorthosilicate (LSO) as a scintillation detector material whereas only 5% (N = 1) use Bismuth Germanate (BGO). In Table 3 and Table 4 the number of devices per examination type is illustrated.

**b) Examination protocols:** In total, data for 463 examination protocols for planar imaging/SPECT and 112 examination protocols for PET were collected. The administered activity was based on a fixed activity value for 87% (N = 401) of the planar/SPECT-examination protocols, whereas for 13% (N = 64) of the protocols administered activity was based on activity per body weight. For PET fixed administered activities were used in 56% (N = 63) of the protocols, while for 44% (N = 49) an activity per body weight approach was used. Table 3 and Table 4 summarize the examination protocols for planar imaging, SPECT and PET/CT examinations together with the mean and median values of the fixed activities and activity per body weight as defined in the protocols for the two activity determination methods, respectively.

**c) Patients:** In total, data for 3,505 individual patients were submitted for planar imaging/SPECT and for 1,045 individual patients for PET. The number (N) of reported values together with the mean weight in kg, mean and median injected activity (in MBq), standard deviation as well as minimum and maximum activity values are reported in Table 3 and 4. Cave: In contrast to the PET examinations where weight was reported for all examinations, for planar imaging/SPECT patient weight was reasonably reported in 96% (N = 3,364) of the patient datasets. In Table 5 the mean and median activity values for patient data from this study (planar/SPECT) are compared to the old NDRL for Austria, NDRL for Switzerland, new NDRL from Germany [37], Croatia and EU. In Table 6 the same is illustrated for PET.

**Table 5:** Planar Imaging/SPECT - Comparison of the median values from this study with published data (NDRL in MBq).

Examination type	Patient data this study mean/median	NDRL Austria old (2010) [19]	NDRL Switzerland (2018) [13]	NDRL Germany (2021) [37]	Proposed NDRL Croatia (2020) [15] (Median)	EU (2014) [14]
Brain imaging: I-123 benzamide, beta-CIT	181/185	185		180		
Salivary gland scan: Tc-99m pertechnetate	162/157	110			400 (369)	
Thyroid imaging: Tc-99m pertechnetate	76/76	110	75	70	200 (185)	80
Thyroid whole body imaging: I-131 sodium iodide	178/185				185 (185)	
Parathyroid imaging: Tc-99m isonitrile	465/400	740	550	550	710 (555)	
Myocardial perfusion imaging: Tc-99m isonitrile 1-day protocol (rest and stress)	1108/1084	1200	1200	total: 1000	1390 (1184)	1200
Myocardial perfusion imaging: Tc-99m isonitrile 2-day protocol (rest and stress)	total: 1085/1000	740 per day	600 per day, 1200 total	400 per application	730 (634)	
Myocardial perfusion/vitality imaging: TI-201 chloride	129/100	110	100			110
Lung perfusion imaging: Tc-99m macroaggregates	128/126	150	180	160	180 (140)	150
Gastric imaging: Tc-99m marked chyme	51/41	110				
Renal imaging: Tc-99m MAG3/EC	101/98	110	100	100	150 (137)	100
Renal imaging: Tc-99m DMSA	109/98	110	120		130 (122)	
Renal imaging: Tc-99m DTPA	222/223	185			150 (130)	
Adrenal imaging: I-123 MIBG	288/260	200				
Bleeding: Tc-99m pertechnetate, erythrocytes	565/556	740	750			
Inflammation imaging: Tc-99m anti-granulocytes antibodies	688/700	740	800		780 (582)	
SNL lymphoscintigraphy: Tc-99m colloid – 1-day protocol	123/98		Total: 80	40	60 (19)	
SNL lymphoscintigraphy: Tc-99m colloid – 2-day protocol	119/136			150		
Bone imaging: Tc-99m bisphosphonate	652/663	740	700	8 MBq/kg	740 (663)	600
Tumour/inflammation/receptor imaging: In-111 somatostatin receptor antagonist	172/160		180	150		
Tumour/inflammation/receptor imaging: Tc-99m somatostatin receptor antagonist	751/740		700	750	740 (740)	
Tumour/inflammation/receptor imaging: Tc-99m PSMA	635/607

**Table 6:** PET - Comparison of the mean and median values from this study with published data (NDRL in MBq).

Examination type	Patient data this study	NDRL Austria old (2010) [19]	NDRL Switzerland (2018) [13]	NDRL Germany (2021) [35]
Brain imaging: F-18 FDG	199/197			3 MBq/kg
Brain imaging: F-18 tyrosine	271/260		210	
Myocardial imaging: F-18 FDG	297/300			
Tumour imaging: F-18 FDG	279/272	400	250	3 MBq/kg
Tumour imaging: Ga-68 somatostatin receptor antagonist	159/162			2.0 MBq/kg
Tumour imaging: Cu-64 PSMA	288/274			
Tumour imaging: F-18 choline	278/272		210	
Tumour imaging: F-18 Fluoride	246/248			
Tumour imaging: Ga-68 PSMA	175/173			2.5 MBq/kg

**Figure 1:**
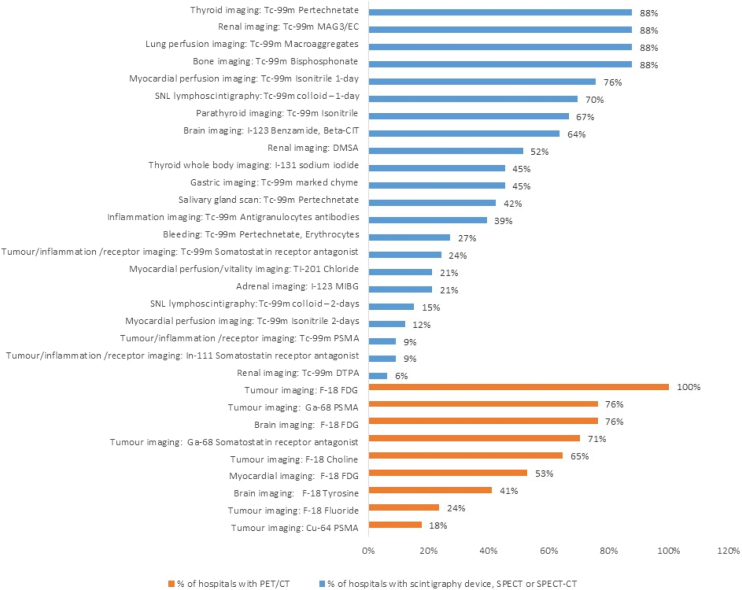
Percentages of hospitals in which examinations are conducted.

The most widely executed examination types for planar imaging/SPECT are bone, thyroid, renal imaging with MAG3/EC and lung perfusion imaging (Figure 1). Data for these examinations was submitted from 88% (N = 29) of all hospitals. The least widely used examination is renal imaging with DTPA receiving data from only 6% (N = 2) of the hospitals. For PET, tumour imaging with FDG is by far the most extensively performed examination (100% of all hospitals with PET/CT; N = 17) whereas tumour imaging with Cu-64 PSMA was documented in only 18% (N = 3) of all hospitals.

**Figure 2:**
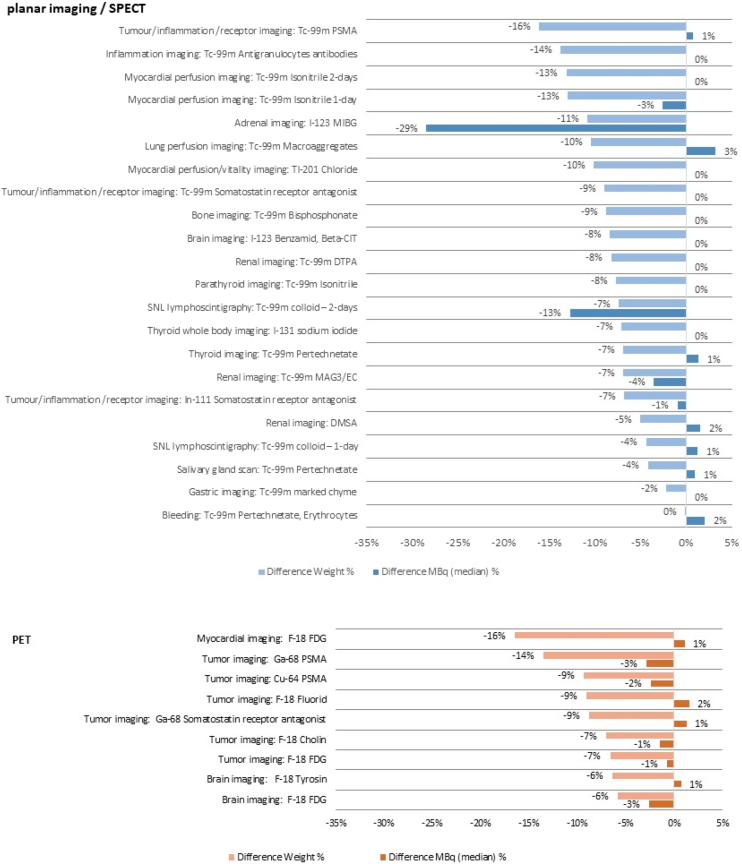
Percentage differences between data without and with (60 kg to 80 kg) weight restriction and impact on median activity per examination type.

ICRP 135 [7] recommends using a weight interval for average sized patients from 60 to 80 kg. To investigate the effects of light and heavy patients on the calculation of the median activities, a new analysis was performed excluding those datasets related to patients weighing less than 60 kg or more than 80 kg (indicated here as “weight-restricted dataset”). Percentage differences between the analysis with all patients and the weight-restricted dataset are shown in Figure 2. With imposing weight restrictions, the overall mean weight decreased from 77 kg to 70 kg for planar imaging/SPECT and from 78 to 71 kg for PET. For planar imaging/SPECT the most impact on mean weight (-16%) was found for Tc-99m PSMA examinations, inflammation imaging (-14%) and myocardial perfusion imaging (-13%). Weight restriction did not impact the findings for “bleeding”. Regarding the differences between median activity, highest difference was found for adrenal imaging with -29%. However, for most of the examination types only minor or no differences were present in the median injected activities after restriction of body weight. Similar could be observed for PET imaging with highest weight differences for myocardial imaging and lowest for brain imaging, however, with associated median activity differences of only 1% to 3%.

To investigate the relationship between different variables a Pearson correlation coefficient was computed. No significant correlation was found between the following variables:

• the year of manufacture of NUC-devices and injected activity (planar imaging/SPECT): Pearson correlation coefficient of r = −0.02, p = 0.14, n = 3649 and PET: Pearson correlation coefficient of r = 0.01, p = 0.87, n = 1045),
• the use of TOF and injected activity (PET: Pearson correlation coefficient of r = −0.03, p = 0.36, n = 845)

**Figure 3:**
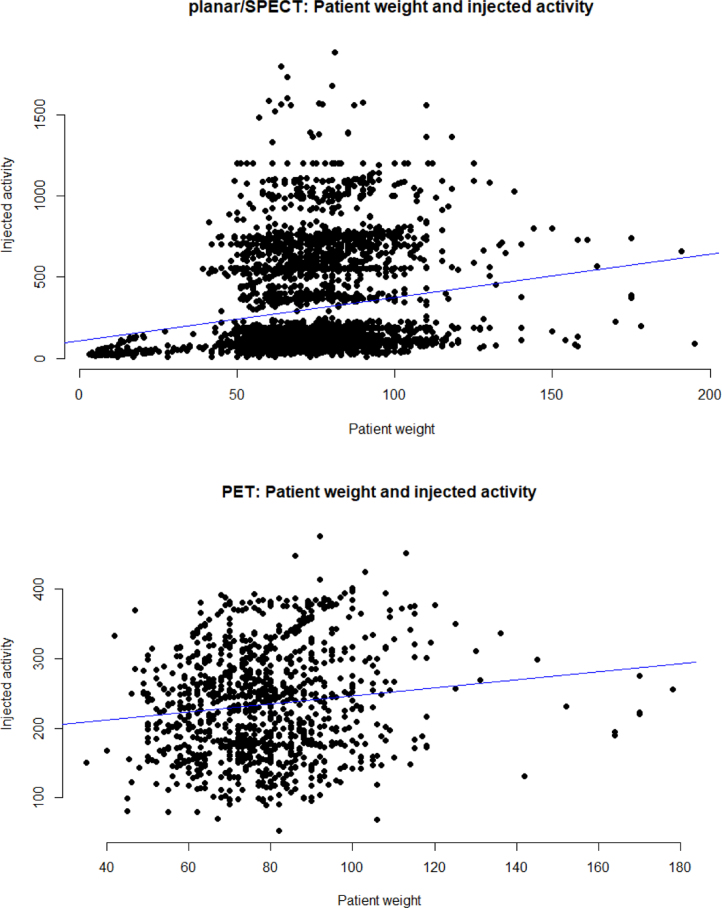
Scatter plots for planar imaging/SPECT and PET comparing patient weight and injected activity.
• Use of IR and the injected activity (PET): Pearson correlation coefficient of r = −0.13, p < 0.01, n = 1045) indicating lower activity values when using iterative reconstruction (negative correlation).
• FOV size and the injected activity (PET): Pearson correlation coefficient of r = −0.09, p < 0.01, n = 1045) indicating lower activity values with larger FOV (negative correlation).

There is a significant correlation between:

• Patient weight and injected activity (planar/SPECT): Pearson correlation coefficient of r = 0.17, p < 0.01, n = 3508 and PET: Pearson correlation coefficient of r = 0.13, p < 0.01, n = 1045) indicating higher activity values for patients with higher weight (positive correlation, Figure 3).

Table 7 shows the NDRL for Austria developed based on this study.

**Table 7:** Austrian NDRL for nuclear medicine as enforced in 2020.

Procedure	Radionuclide and radiopharmaceutical	NDRL [MBq]
*Planar imaging/SPECT*		
Brain imaging	I-123 ioflupane	185
Salivary gland scan	Tc-99m pertechnetate	110
Thyroid imaging	Tc-99m pertechnetate	75
Thyroid whole body imaging	I-131 sodium iodide	185*
Parathyroid imaging	Tc-99m isonitrile	460
Myocardial perfusion imaging	Tc-99m isonitrile 1-day protocol	total: 1100
Myocardial perfusion/vitality imaging	TI-201 chloride	100
Lung perfusion imaging	Tc-99m macroaggregates	130**
Renal imaging	Tc-99m MAG3/EC and Tc-99m DMSA	110
Adrenal imaging	I-123 MIBG	200
Bleeding	Tc-99m pertechnetate, erythrocytes	740
Bone imaging	Tc-99m bisphosphonate	650

*PET*		
Brain imaging	F-18 FDG	180
Brain imaging	F-18 tyrosine	250
Myocardial imaging	F-18 FDG	300
Tumour imaging	F-18 FDG	280
Tumour imaging	F-18 choline	250
Tumour imaging	Ga-68 somatostatin receptor antagonist	150
Tumour imaging	Ga-68 PSMA	150

Comments:

* 370 MBq in patients with thyroid stimulation.

** for previous inhalation.

## Discussion

This study summarizes the outcome of a recent survey of the employed activity levels in nuclear medicine procedures in Austria with the aim to provide a well-founded basis for the update of the NDRL. The study design was tailored to achieve a maximum response rate to enable a valid assessment of the used dosages given the comparatively low absolute number of nuclear medicine departments in Austria. This was successfully achieved for in hospital nuclear medicine departments with a response rate of 100%.

The collected dose data are in general comparable to those found in other recent studies (Table 5 and Table 6) [13], [14], [15], [35], [38]. This is reasonable as today several international guidelines and recommendation for most of the standard examinations exist, which were developed in multilateral consensus between the national professional societies (e.g. [39], [40], [41]). However, compared to the, at the time of the survey valid NDRL in Austria, deviations of >20% in average activity were found for nearly half (8/17) of the surveyed procedures (Table 5 and 6). For most of the examinations with deviation of >20%, injected activities were found to be lower than the NDRL. For example, the average injected dose for tumour imaging with F-18-FDG was found to be 30% below the NDRL. This is expected to result from technological improvements, such as 3D PET data acquisition, IR and increased sensitivities.

During the data analysis also different reasons for deviating injected activities where found. For some of the radiopharmaceuticals the NDRL valid at the time of the survey where not in agreement with the dose recommendation for the radiopharmaceuticals. For example, Thyroid imaging using Tc-99m pertechnetate was done on (median) injecting 76 MBq and parathyroid imaging using Tc-99m isonitrile using (median) 400 MBq (Table 5). Both median activities were below the NDRL of 110 MBq and 740 MBq, respectively. However, the injected activities were in agreement with the recommended dosage as defined in the pharmaceutical information leaflet of 20-80 MBq and 200-700 MBq, respectively. Such inconsistencies in prescribed doses and NDRL should be avoided. Thus, prescribed doses need to be reviewed and taken into account during updates of NDRL.

In general, the results nicely show the necessity to update DRL on a regular basis to account for changes in clinical practice and technological advances. As seen in the correlation analysis, especially IR and increased sensitivity due to extended FOVs in PET devices seemed to contribute to a general dose reduction. While being reasonable as such, it is also in line with findings for the use of IR in CT [34]. Another interesting finding pertains to the correlation of administered activities with weight. Although, a general significant correlation of patient weight with dose was found, a re-evaluation of the data in a subset of standard patients with weights of 70 ±10 kg revealed no practically considerable changes in injected activity levels for most of the protocols (Figure 2). The reason therefore is expected to be the relatively high number of protocols with fixed activity levels (87%). This facilitates the derivation of DRL as deviations of data reported for individuals from a standard patient do not influence the general average doses. However, rigor data inspections and weight restrictions might be necessary to derive reasonable DRL in cases where patient weight-based dosage is used in the majority of sites.

## Limitations

To keep the workload for participating hospitals reasonable and to achieve a high participation rate, we limited the minimum number of patient datasets per examination type to 10. This approach was also used in previous studies [33], [34] and is regarded as appropriate, if the participation rate is high. As NUC examinations are more standardised than radiological examinations and all hospitals with NUC equipment participated, we believe the data quality in this work represents a good compromise between theoretical optimum and practicability of the data collection.

## Conclusion

This work presents data on injected activities used in clinical practice for diagnostic nuclear medicine procedures in Austria to provide a base for the update of the NDRL. The injected activities found are comparable with those reported in other countries. However, for procedures for which NDRL exist, deviations in injected activities of >20% compared to the NDRL were found. Reasons therefore are assumed to result mainly from advances in technology but also from inconsistencies between NDRL and prescribed dosages as given in the information leaflets of the radiopharmaceuticals.

## Funding

This study has received funding by the Austrian Federal Ministry of Health.

## Conflict of interest

Michael Hinterreiter is an employee of GE Healthcare Handels GmbH (Technologiestraße 10, 1120 Vienna, AUT). The other authors declare that they have no conflict of interest.
